# Molecular Insight into Human Lysozyme and Its Ability to Form Amyloid Fibrils in High Concentrations of Sodium Dodecyl Sulfate: A View from Molecular Dynamics Simulations

**DOI:** 10.1371/journal.pone.0165213

**Published:** 2016-10-21

**Authors:** Majid Jafari, Faramarz Mehrnejad

**Affiliations:** Department of Life Sciences Engineering, Faculty of New Sciences & Technologies, University of Tehran, Tehran, Iran; Jamia Millia Islamia, INDIA

## Abstract

Changes in the tertiary structure of proteins and the resultant fibrillary aggregation could result in fatal heredity diseases, such as lysozyme systemic amyloidosis. Human lysozyme is a globular protein with antimicrobial properties with tendencies to fibrillate and hence is known as a fibril-forming protein. Therefore, its behavior under different ambient conditions is of great importance. In this study, we conducted two 500000 ps molecular dynamics (MD) simulations of human lysozyme in sodium dodecyl sulfate (SDS) at two ambient temperatures. To achieve comparative results, we also performed two 500000 ps human lysozyme MD simulations in pure water as controls. The aim of this study was to provide further molecular insight into all interactions in the lysozyme-SDS complexes and to provide a perspective on the ability of human lysozyme to form amyloid fibrils in the presence of SDS surfactant molecules. SDS, which is an anionic detergent, contains a hydrophobic tail with 12 carbon atoms and a negatively charged head group. The SDS surfactant is known to be a stabilizer for helical structures above the critical micelle concentration (CMC) [1]. During the 500000 ps MD simulations, the helical structures were maintained by the SDS surfactant above its CMC at 300 K, while at 370 K, human lysozyme lost most of its helices and gained β-sheets. Therefore, we suggest that future studies investigate the β-amyloid formation of human lysozyme at SDS concentrations above the CMC and at high temperatures.

## Introduction

Protein folding is one of the great unsolved biological problems in basic and applied sciences [2–5]. The exploration of the mechanism of protein folding establishes an understanding of the relationship between protein structure and stability and identifies factors that affect native protein conformational stability. The investigation of the regulation of unfolding is considered useful for understanding protein folding mechanisms. From a biological sciences perspective, the study of protein unfolding provides the necessary background for enhancing the stability of biocatalysts used in biological and biomedical processes and for carrying out these processes in different solutions [6, 7]. Thus, to gain a fundamental understanding of folding and stability of proteins, the mechanism of action of surfactants, among other compounds, must be studied. These studies should focus on solvation, energetic features, and the structures of nonnative proteins at detailed levels comparable with those that have been achieved for native proteins [8, 9]. Previous studies have shown that the interactions between ionic surfactants and oppositely charged proteins could be very strong [10–13]. Ionic surfactants typically bind to proteins through a complex of hydrophobic and electrostatic interactions [14]. Ionic surfactants with high affinity for proteins indicate that surfactant molecules can bind to proteins in monomeric states and as micellar systems. Typically, the binding of monomeric surfactants only leads to local changes in protein conformation, while global and cooperative unfolding occurs around the critical micelle concentration (CMC) [15]. Therefore, the effect of surfactants, such as sodium dodecyl sulfate (SDS), on the folding and unfolding of a protein depends on the concentrations of SDS and the protein as well as the nature of the protein [16]. Micelle-protein interactions are complicated and play key roles in the unfolding of proteins. The interactions of surfactants with proteins have been widely studied due to their industrial and medical applications [17–19]. However, the mechanisms by which surfactants influence protein structure are not well understood. In this work, SDS was studied due to its wide use in the characterization, separation and purification of proteins [20–24]. Unlike other denaturants, such as urea or guanidine hydrochloride, which are effective only at molar concentrations [25–27], SDS is effective at millimolar concentrations [25–27]. Additionally, SDS is a strongly denaturing anionic surfactant for many proteins and is widely used in biological applications to solubilize proteins in a denatured state. Previous studies have shown that SDS molecules bind to most proteins with high affinity. The mechanism of denaturation by SDS depends on the secondary structure of the protein; β-sheet proteins are more resistant to unfolding than α-helical or mixed α/β proteins [28]. SDS molecules can bind to proteins via interactions between sulfate groups and the side chains of positively charged residues and likewise between alkyl chains and hydrophobic side chains.

In this study, human lysozyme is used as a model protein to investigate folding and unfolding processes and protein-surfactant interactions [29–31]. From a medical point of view, the significance of folding problems has recently increased because protein aggregation has been shown to lead to a number of fatal diseases, such as lysozyme systemic amyloidosis [31–35]. Systemic amyloidosis is a lysozyme-associated disease caused by the deposition of lysozyme in the amyloid fibril form in spleen, kidney, and liver cells [33, 36]. Previous studies have shown that lysozyme can easily form amyloid fibrils under different conditions, such as high temperatures and appropriate pH conditions [37–40]. Many amyloidogenic proteins interact with the surfaces of basement membranes; SDS has the potential to mimic this condition [29, 41–45]. Furthermore, SDS surfactants mimic the bio-membrane environment and can provide anionic conditions through their anionic head groups [42, 46]. Previous experimental studies revealed that SDS has a great tendency to induce amyloid fibril formation in some classes of proteins [47–50]. Therefore, an investigation on the molecular behavior of human lysozyme in the presence of SDS surfactant is of great importance.

Lysozyme is an antibacterial enzyme that exists in external secretions [51] and has a length of 130 amino acids and a molecular mass of 14.3 kDa. It belongs to the α+β class of proteins and contains 11 anionic amino acid residues and 19 cationic residues. Furthermore, this protein is positively charged in aqueous solutions. Lysozyme is a stable protein due to its four disulfide bonds (C6-C128, C30-C116, C65-C81, C77-C95), and its surface is mostly polar, whereas its interior is almost always hydrophobic [30]. A few classes of surfactants, such as SDS, in specific ambient conditions, can denature lysozyme. This behavior makes lysozyme an excellent model for investigating protein—surfactant interactions. The aims of the present study were to explore the amyloid formation or aggregation of human lysozyme in the presence of SDS molecules upon temperature change and to further the molecular insight into all interactions between SDS-lysozyme complexes. Molecular dynamics simulations of human lysozyme in aqueous SDS solutions were compared with simulations using water to determine the net effect of the ionic surfactant on human lysozyme conformations. Therefore, the discussion section focuses on the interactions between the model protein and SDS molecules.

## Computational Section

### Computational Methods

**Fig 1:**
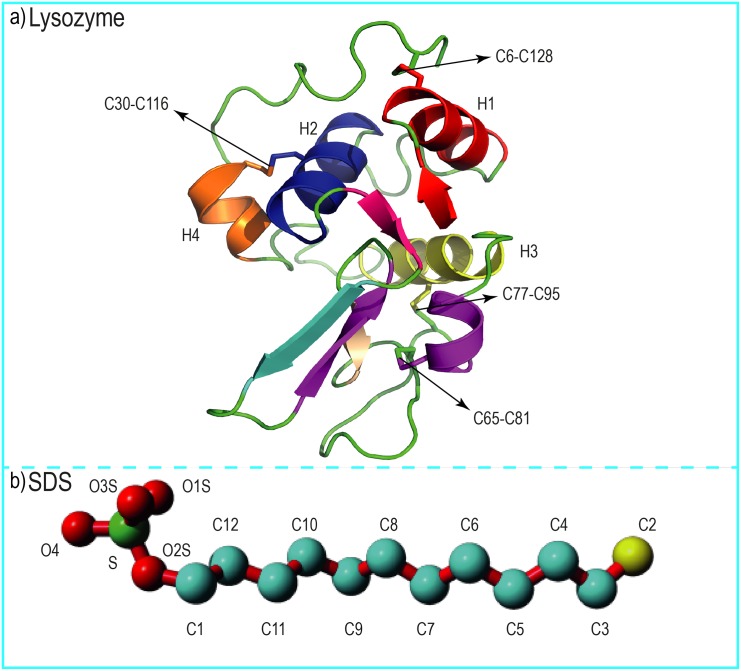
The structures of human lysozyme and sodium dodecyl sulfate. (a) The native structure of human lysozyme represented as a new cartoon model. The α- helix structures are shown with the letter H and C6-C128, C30-C116, C65-C81, and C77-C95 represent disulfide bounds in the human lysozyme structure. (b) The structure of an SDS surfactant molecule with its polar head group (in red and green) and hydrophobic tail (in cyan and yellow) is shown as a ball and stick model.

MD simulations and subsequent analyses were carried out with the GROMACS simulation package, version 4.5.6. The initial conformation for the simulations of lysozyme was obtained from the Protein Data Bank (PDB ID: 1LZ1) [30] (Fig 1). The starting coordinates of SDS were taken from works carried out by Sammalkorpi et al. [52]. The protein was solvated in a cubic box large enough to contain the protein and 1 nm of solvent on all sides using the SPC216 water model [53] and a mixture of SDS and water. All systems were neutralized by adding Cl and Na counter-ions. The systems were initially energy minimized through a total of 3000 steps of calculations: position-restrained steepest descents, steepest descents and the conjugate gradient method. The short-range electrostatic interactions were calculated at 1.0 nm. The Particle Mesh Ewald (PME) algorithm was used to treat long-range electrostatic interactions. The Lennard-Jones potential was applied to calculate the van der Waals interactions [54]. The LINCS algorithm was applied to constrain bond lengths, allowing for a time step of 2 fs for all MD systems.

The pressure and temperature were kept at reference values (P = 1 bar, T = 300 or 370 K) using Berendsen-type pressure coupling at 1 bar with a coupling coefficient of τP = 0.1 ps and Berendsen-type temperature coupling with a coupling coefficient of τT = 0.1 ps. To avoid void formation, each system was gradually heated from 50 K to the desired temperature over 200 ps in a canonical (NVT) ensemble on the proteins. Subsequently, the whole system (the protein, the SDS molecules and the solvent) was equilibrated in an isothermal—isobaric (NPT) ensemble without any restraints on the protein for 1 ns at one atmospheric pressure. Following an overall 100 ps equilibration time, the production runs were carried out on the NPT ensemble at one atmospheric pressure. The lengths of the final production MD simulations are reported in Table 1.

**Table 1:** Summary of MD simulations with details ^a^.

System	Protein	Number of water molecules	Number of SDS/Na+ molecules	Box volumes (nm^3^)	Temperature (K)	Duration
Water_300	Lysozyme	9442	-	300.65	300 K	500*2 ns
Water_370	Lysozyme	9442	-	300.65	370 K	500*2 ns
SDS_300	Lysozyme	8925	200/200	300.65	300 K	500*2 ns
SDS_370	Lysozyme	8925	200/200	300.65	370 K	500*2 ns

^a^ Each system is indicated as follows: Water-300 (human lysozyme in pure liquid water at 300 K), Water-370 (human lysozyme in pure liquid water at 300 K), SDS-300 (human lysozyme in aqueous SDS solution systems at 300 K), SDS-370 (human lysozyme in aqueous SDS solution systems at 370 K). All simulations are run for 500000 ps (500 ns).

### Calculation of Binding Free Energy

To further understand the interactions in the lysozyme-SDS complex, binding free energies were calculated with the Molecular Mechanics-Poisson Boltzmann Surface Area (MM-PBSA) method using g_mmpbsa tools [55, 56]. The binding free energies for the lysozyme and the SDS surfactant were obtained using the following equation (Eq 1):

$$\Delta G_{binding}=\;G_{complex}-\left(G_{lysozyme}+\;G_{SDS}\right)$$ (1)

where G_complex_ is the total free energy of the lysozyme-SDS complex, G_lysozyme_ is the total free energy of the lysozyme, and G_SDS_ is the total free energy of the SDS surfactant in solvent.

The free energy per residue that contributed toward the total binding free energy was calculated as (Eq 2)

$$G=<E_{MM}>+<G_{solvation}>-TS$$ (2)

where E_MM_ is the average molecular mechanics potential energy obtained based on the GROMOS96 53a6 molecular mechanics force-field in a vacuum, G_solvation_ is the free energy of solvation, and T and S are temperature and entropy, respectively.

The entropy contribution (TS) of the lysozyme was neglected because the purpose of this study was to calculate the contribution of each residue to the binding free energies.

E_MM_ was obtained using (Eq 3)

$$E_{MM}=\;E_{b}+\;E_{nb}$$ (3)

where E_b_ is the bonding interactions and E_nb_ is the non-bonding interactions, including electrostatic and van der waals interactions (Eq 4)

$$E_{nb}=\;E_{electrostatic}+\;E_{van\;der\;waals}$$ (4)

E_electrostatic_ and E_van der waals_ were obtained using the Coulomb and Lennard-Jones (LJ) potential functions, respectively. A single-trajectory approach was used to calculate the free energy, i.e., E_nb_ was set to zero.

The free energy of solvation was calculated with the following equation (Eq 5):

$$G_{solvation}=\;G_{pols}+\;G_{npols}$$ (5)

where G_pols_ is the electrostatic part of the free energy of solvation and G_npols_ is the non-electrostatic part of the free energy of solvation. To calculate G_npols_, the SASA-only non-polar model was used, since the SASA model is widely used to calculate non-polar solvation energy [55].

The non-polar solvation free energy was calculated using the following equation (Eq 6):

$$G_{npols}=\;\gamma x+b$$ (6)

where γ is the surface tension coefficient of the solvent, x is the SASA, and b is constant. To calculate the binding free energies in the SDS-lysozyme complexes in each MD simulation, 100 snapshots were extracted from the final 40000 ps trajectory.

## Results

### Protein Conformation

**Fig 2:**
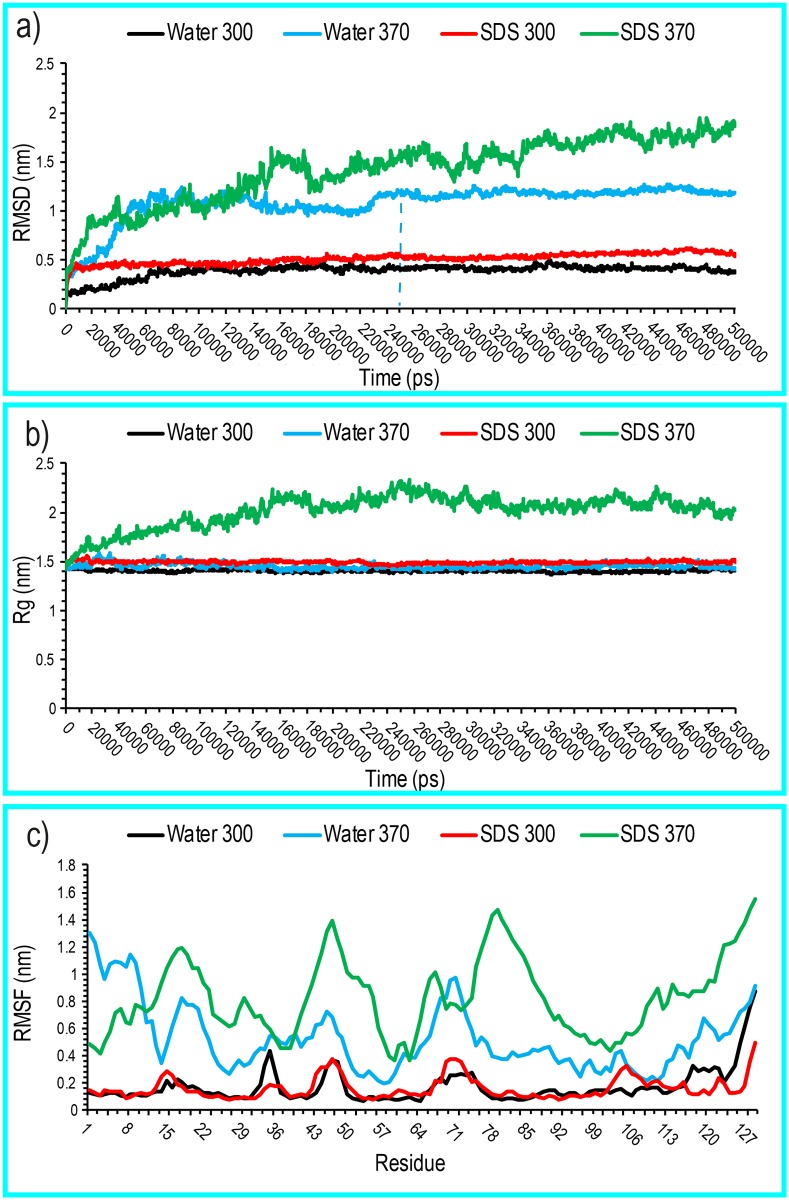
The Cα RMSD and the radius of gyration of human lysozyme. (a) and (b) represent the time evolution of the Cα RMSD and the radius of gyration of human lysozyme in pure liquid water and aqueous SDS solution at 300 K and 370 K, respectively. (c) represents the RMSF of Cα atoms as a function of residue number.

The time dependence of the C_alpha_ root mean square deviation (RMSD) provides information on the structural stability of proteins. Therefore, the first step in quantifying the effect of SDS on the structure and dynamics of lysozyme is the investigation of the RMSD of the initial conformation. In the aqueous protein solution at 300 K, human lysozyme had an average RMSD value of approximately 0.39 nm, suggesting that the structure of the protein largely maintained its native conformation in pure liquid water at this temperature. In the SDS simulation at 300 K, the RMSD for lysozyme reached approximately 0.5 nm. The RMSD results at 370 K revealed that lysozyme began to unfold and continued to gradually unfold as the temperature increased. However, in pure liquid water, the RMSD reached a plateau after a simulation of approximately 250000 ps simulation, which indicated that the lysozyme nearly reached a stable structure (dashed line Fig 2a). These data show that lysozyme is more stable in pure liquid water and aqueous SDS simulations at 300 K than in other simulations at 370 K (Fig 2a). As a result, when compared with pure water simulations, SDS molecules seem to destabilize the native structure of human lysozyme at high temperatures. Thus, SDS molecules seem to play a negative role in affecting the structural integrity of globular proteins at increased temperatures.

**Fig 3:**
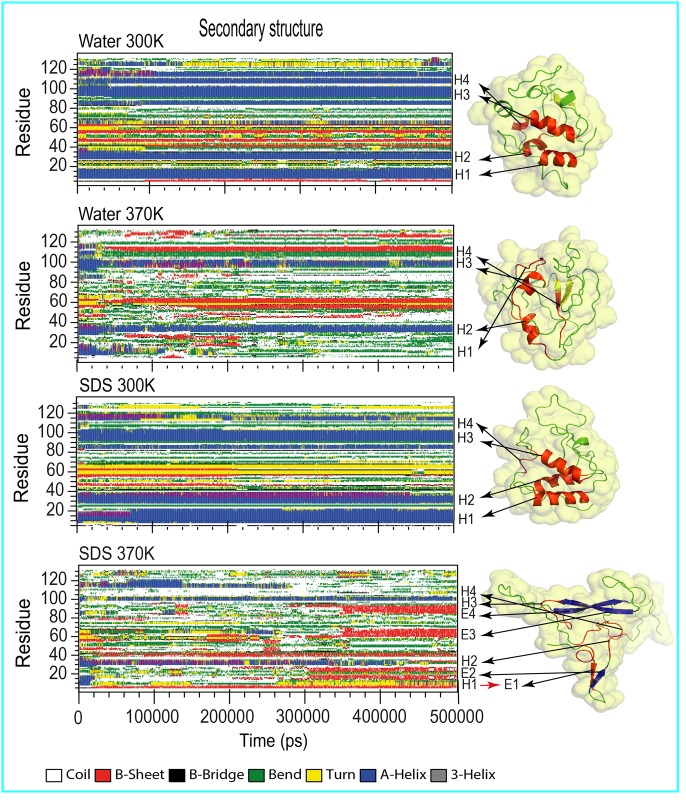
Human lysozyme secondary structure analysis with DSSP. Left: time evolution of the secondary structure of human lysozyme through DSSP in pure liquid water or aqueous SDS solution at 300 K and 370 K. Right: a snapshot of human lysozyme extracted from last step trajectory (500000 ps) in each MD simulation. Helix and β-sheet structures are shown with the letters H and E, respectively.

The radius of gyration is useful for measuring the compactness of a protein structure. Hence, the radius of gyration (Rg) of human lysozyme was also calculated (Fig 2b). Previous experimental studies revealed that at temperatures below the thermal denaturation midpoint (Tm) of lysozyme and in the presence of SDS above its CMC, lysozyme maintained its helicity [57] but not its β-sheet structure [46]. As shown in Fig 2b, in the SDS 300 K simulation, the Rg was greater than those of the pure water MD simulations at 300 K and 370 K. This could be due to a number of lysozyme secondary structures, such as β-sheet structures, becoming denatured and thereby causing the lysozyme to lose its native compactness. The Rg of pure water at 370 K in the first 150000 ps simulation showed several peaks, which were higher than those of SDS at 300 K, and reached a plateau below the Rg of SDS at 300 K. This was because the H1 and H4 helices and portions of the H2 and H3 helices lost their helical structures, and portions of the H4 helix and bend structures gained β-sheet structures due to the increased compactness of human lysozyme (Fig 3). The Rg values drastically increased in the aqueous SDS solution at 370 K. These results indicated that the Rg value increased more significantly in the aqueous SDS solution at 370 K than in the other MD simulations (Fig 2b). In other words, the structural changes of lysozyme were the greatest in the aqueous SDS solution at 370 K. Furthermore, the changes in the structural compactness of lysozyme were smallest in pure water at both temperatures and in the aqueous SDS solution at 300 K.

Additional data on the observed conformational changes were calculated from the root mean square fluctuation (RMSF) of the C_alpha_ atoms (Fig 2c) and the time evolution of the secondary structure elements (Fig 3). The MD simulations indicated that the RMSF per residue of the overall mobility of the residues in the aqueous SDS solution at 370 K was larger than those of the other simulations (Fig 2c). The RMSF values for residues 69–73 and 1–13 were lower than those of the pure water 370 K simulation. This may be due to the higher stability of the C6-C128 and C65-C85 disulfide bounds in the SDS surfactant than that of the other disulfide bounds, which were not observed in water at 370 K during the 500000 ps simulation. An important observation is that in the all-unfolding simulations, the protein was rapidly destabilized in the SDS solution at 370 K.

Fig 3 shows variations in the secondary structure of the protein during the simulations [54]. In agreement with experimental studies [30], the analysis of the structure of human lysozyme in water at 300 K showed that the lysozyme consisted of 44.5%, 8%, and 24% α-helix, β-sheet, and turn conformations, respectively (Table 2). After 20000 ps in the pure liquid water MD simulation at 370 K and during the early stage of the SDS 370 K simulation, the H4 helix completely unfolded (Fig 3). In the presence of SDS at 300 K, our analyses showed that the H1, H2, and H3 helical structures were nearly fully stabilized during the MD simulation. In the aqueous SDS solution at 300 K, the contents of the β-strands denatured with increasing simulation time, whereas the α-helices were stably maintained during the simulation (Fig 3). At 370 K in the SDS aqueous solution, the H1, H2, and H4 helices were entirely disrupted, the H3 helix was partially disrupted and the H1 helix and specific portions of lysozyme were converted to β-sheet structures. In pure water at 370 K, H2 and H3 were somewhat maintained, but the H1 helix was entirely disrupted, and the H4 helix was converted to a β-sheet structure. At 370 K, the SDS simulation indicated more atomic fluctuations in the whole protein than in the water simulation. As shown in Fig 3, SDS exhibited the ability to destabilize the secondary structures of the lysozyme at higher temperatures.

**Table 2:** The percentages of average secondary structure for Lysozyme during the 500 ns MD simulation.

System	Protein	Temperature (K)	Average secondary structure content (%)		
			α-helix	β-sheet	Other
Lysozyme [30] ^a^	Lysozyme	-	39	7	52
Water_300	Lysozyme	300 K	35	8	57
Water_370	Lysozyme	370 K	10	13	77
SDS_300	Lysozyme	300 K	30	3	67
SDS_370	Lysozyme	370 K	7	13	80

^a^ Percent referenced to the x-ray structure.

### Direct Interaction Between the Proteins and Solvent

**Fig 4:**
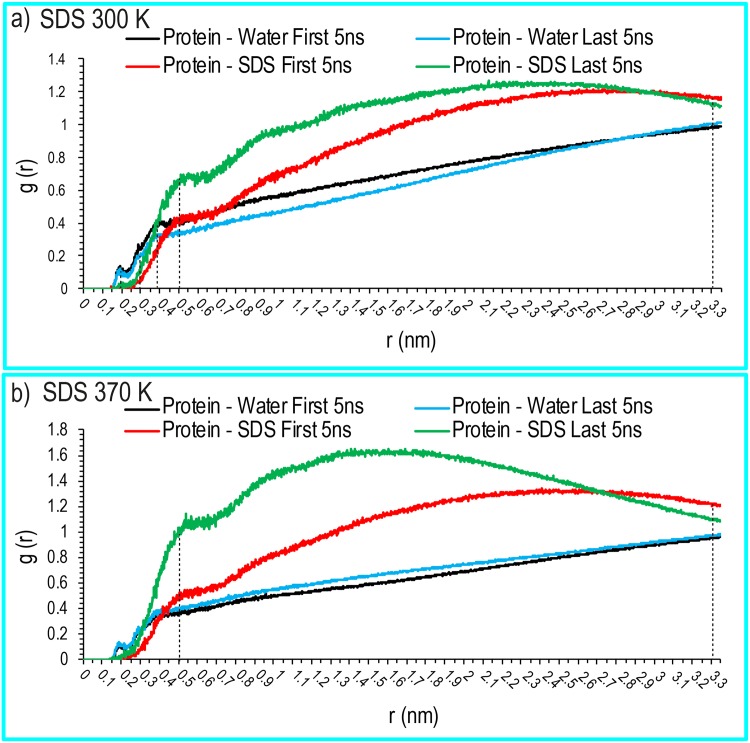
Radial distribution functions for human lysozyme. Represents the radial distribution functions for human lysozyme in contact with water or SDS molecules in pure liquid water and the aqueous SDS solution systems at 300 K (a) and 370 K (b).

**Fig 5:**
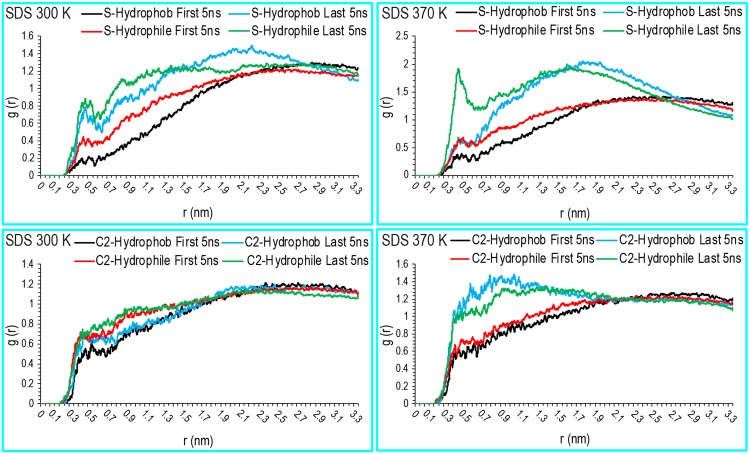
Radial distribution functions for hydrophobic and hydrophilic residues of human lysozyme. Represents the radial distribution functions for hydrophobic and hydrophilic residues of human lysozyme touching the C2 and S atoms of SDS in the aqueous SDS solution systems at 300 K and 370 K.

Regarding the structure of the water and SDS molecules around the protein, we first calculated the RDF of water around the lysozyme molecules in all systems. Fig 4 shows the distribution of water and SDS molecules around human lysozyme. A first peak was observed at approximately 0.20 nm, which corresponded to the first hydration shell. A second peak was observed at approximately 0.30 nm, which suggested hydrogen bonding interactions between the proteins and the water (dashed lines in Fig 4). A more pronounced third peak appeared at 0.4 nm, which implied the presence of non-hydrogen-bonding interactions. Protein hydration patterns at 370 K were qualitatively similar to those at 300 K in the lysozyme simulations. These results showed that high densities of SDS molecules were distributed around the proteins at approximately 0.50 nm to 3.30 nm from the center of mass (COM) of the lysozyme at 300 and 370 K. This indicated that a large number of SDS molecules distributed around the proteins and expelled water molecules from the protein surface. Strong direct interactions between the SDS molecules and the proteins can be shown to induce the denaturation of human lysozyme. To study the orientation of SDS molecules surrounding the proteins, the RDF values of the SDS molecules with respect to the hydrophobic and hydrophilic amino acids were calculated. In all simulations, SDS sulfate groups were oriented toward hydrophilic amino acids, including those with positively charged side chains (Fig 5).

**Fig 6:**
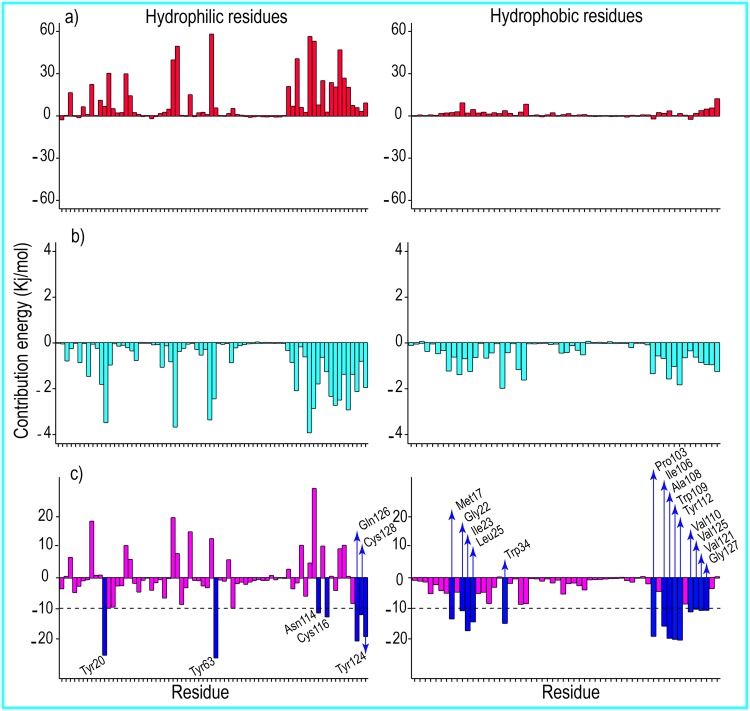
The contribution energy per residue in the total binding free energies 1. (a) Polar contribution of free energy per residue, (b) non-polar contribution of free energy of each residue, and (c) total contribution of free energy of per residue to the formation of the human lysozyme-SDS complex at 300 K. Left, hydrophilic residues; right, represent hydrophobic residues of human lysozyme. The critical residues for the formation of the human lysozyme-SDS complex (residues with ΔG_b_ < -10 Kj/mol (< -2.4 Kcal/mol)) are labeled and shown with blue bars.

**Fig 7:**
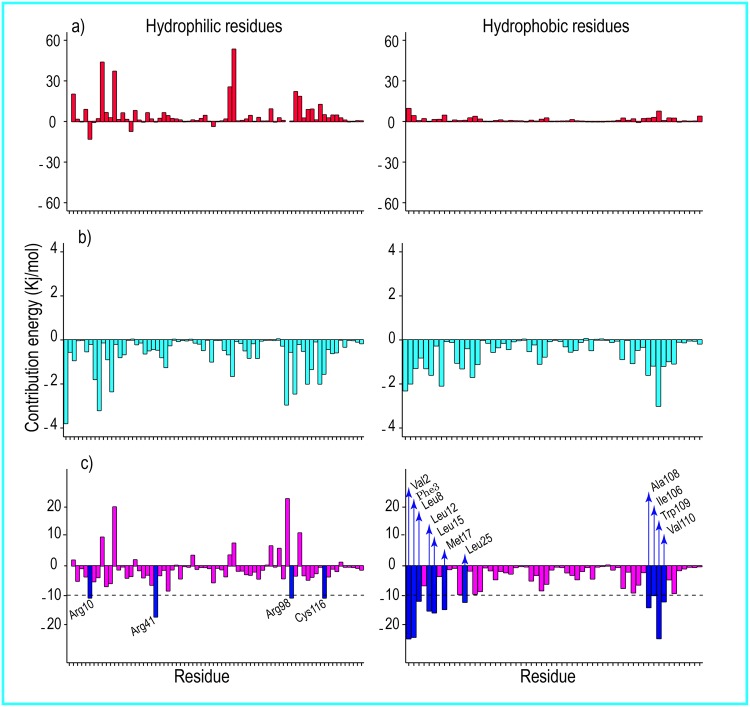
The contribution energy per residue in the total binding free energies 2. (a), (b), and (c) represent the polar, non-polar, and total contributions of free energy of per residue, respectively to the binding free energy at 370 K. Left, hydrophilic residues; right hydrophobic residues of human lysozyme. The critical residues for the formation of the human lysozyme-SDS complex (residues with ΔG_b_ < -10 Kj/mol (< -2.4 Kcal/mol)) are labeled and shown with blue bars. The contribution of free energy for Lys-1 in a and c is not shown because of its high values ΔG_pb_ < 201.05 Kj/mol and ΔG_b_ < 72.17 Kj/mol.

Most SDS C2 atoms were expected to be oriented toward hydrophobic patches; however, orientations toward both protein hydrophilic and hydrophobic patches were observed. These results were confirmed by calculating the contribution energy per residue in the total binding free energies (Figs 6 and 7). As seen in Figs 6b and 7b, most of the hydrophilic residues at 300 K had more non-polar interactions with the SDS molecules than those at 370 K (residues with ΔG_npb_ < -2 Kj/mol; further details are provided below). This suggested that the hydrophobic tails of the SDS molecules surrounded the lysozyme molecules.

### Average of Total Binding Free Energy

**Fig 8:**
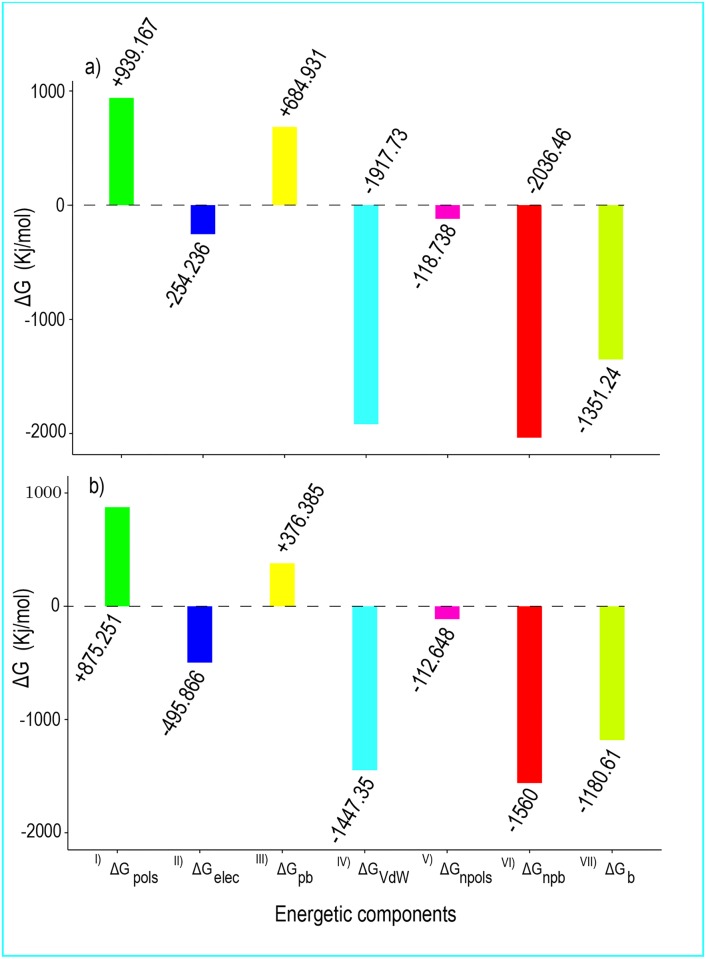
Energetic components of the human lysozyme-SDS complex. All energetic components of the human lysozyme-SDS complex at (a) 300 K and (b) 370 K during the last 40000 ps of the MD simulation. (I) is polar solvation energy, (II) is electrostatic energy, (III) is polar binding energy, (IV) is van der Waals energy, (V) is non-polar solvation energy (SASA energy), (VI) is non-polar binding energy, and (VII) is Binding energy.

Fig 8 shows the averages of the binding free energies and their corresponding components in the lysozyme-SDS complexes at 300 K and 370 K.

The polar binding free energy, non-polar binding free energy, and total binding free energy are respectively expressed as (Eqs 7–9)

$$\Delta G_{pb}=\;\;\Delta G_{pols}+\;\Delta G_{electrostatic}$$ (7)

$$\Delta G_{npb}=\;\Delta G_{vdw}+\;\Delta G_{npols}$$ (8)

$$\Delta G_{b}=\;\Delta G_{pb}+\;\Delta G_{npb}$$ (9)

**Fig 9:**
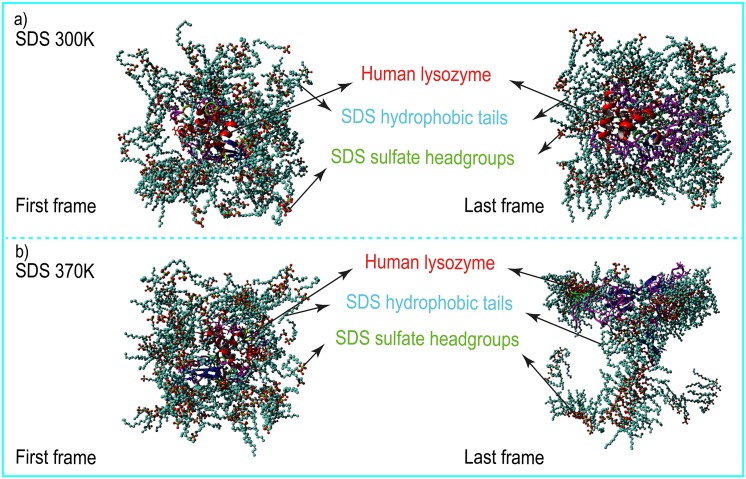
Orientation of SDS surfactants around human lysozyme. (a) and (b) show the first and the last frames of the trajectory in two ambient conditions (aqueous SDS solution at 300 K (upper) and 370 K (lower)).

The results in Fig 8 showed that ΔG_pb_ (684.931 kJ/mol) was unfavorable and that ΔG_npb_ (-2036.463 kJ/mol) was favorable for the formation of the lysozyme-SDS complexes. This suggested that non-polar interactions have essential roles in the formation of the complex. Furthermore, the lysozyme molecules were mostly surrounded by the hydrophobic tails of the SDS molecules. Fig 9 shows four images extracted from the first and last steps of the trajectory files of the MD simulations to show how the SDS surfactant molecules surrounded human lysozyme.

### Contribution Per Residue to the Binding Free Energies

The polar, non-polar, and total interaction contributions of each residue were calculated (Figs 6 and 7). As seen in Figs 6 and 7 (panels (a) and (b)), the hydrophilic and hydrophobic residues generally had hydrophobic interactions and no polar interactions with the SDS surfactants at both studied temperatures. Furthermore, with respect to Figs 6c and 7c, the hydrophobic residues had major roles in the absorption of SDS on human lysozyme at both temperatures. These results suggested that the hydrophobic and hydrophilic residues of human lysozyme were surrounded by the hydrophobic tails of the SDS molecules. The polar binding interaction energies for the Lys-1 and Arg-41 residues at 300 K were -2.59 and -1.77 kJ/mol, respectively. The polar binding interaction energies for the Arg-10, Lys-33, and Arg-62 residues at 370 K were -12.92, -7.17, and -3.54 kJ/mol, respectively. The residues required for the formation of interactions in the lysozyme-SDS complexes at both temperatures are listed in Table 3.

**Table 3:** The critical residues for the absorption of SDS on lysozyme, based on their total binding free energy contributions (residues with ΔG < -10 Kj/mol (< -2.4 Kcal/mol)) ^a^.

Residues	SDS 300 K	SDS 370 K
Hydrophilic residues	Tyr-20, Tyr-63, Asn-114, Cys-116, Tyr-124, Gln-126, Cys-128	Arg-10, Arg-41, Arg-98, Cys-116
Hydrophobic residues	Met-17, Gly-22, Ile-23, Leu-25, Trp-34, Pro-103, Ile-106, Ala-108, Trp-109, Val-110, Tyr-112, Val-121, Val-125, Gly-127	Val-2, Phe-3, Leu-8, Leu-12, Leu-15, Met-17, Leu-25, Ile-106, Ala-108, Trp-109, Val-110

^a^ Each amino acid is shown with its three-letter codes. The total binding free energy ΔG_b_ is obtained using Δ*G*_*b*_ = Δ*G*_*pb*_+Δ*G*_*npb*_ (Eq 9). Where ΔG_pb_ is the polar binding free energy and ΔG_npb_ is the non-polar binding free energy.

## Discussion

For many years, proteins have been characterized using SDS polyacrylamide gel electrophoresis (PAGE). SDS is an anionic detergent and contains a hydrophobic alkyl tail and a negatively charged head group. Most proteins contain negatively and positively charged amino acids. Therefore, ionic surfactants readily bind to proteins with high affinity. The interactions of proteins, especially lysozyme, with SDS have been extensively studied using a variety of experimental and theoretical methods [31, 58–62]. These works have suggested that the interaction of SDS with proteins is relatively complicated. In the presence of SDS molecules at 370 K, the hydrophobic core of the protein was exposed to solvent, leading to global and cooperative unfolding. Yonath et al. showed that SDS molecules tended to interact with the interior domain of lysozyme rather than at the protein surface, resulting in conformational changes in which the global structure of the protein is affected. However, a large portions of lysozyme maintains its conformation even in the presence of SDS [63]. In aqueous SDS solutions, proteins with higher contents of β-sheets were particularly resistant to unfolding compared with proteins with higher contents of α-helices [64].

SDS has been shown to be capable of providing biological membrane-like environments. Amyloidogenic proteins are known to interact with biomembranes [42, 46, 65]. Two amyloidogenic variants of human lysozyme (Ile56Thr and Asp67His) and their wild-type forms are known as amyloidogenic proteins [66]. Therefore, the investigation of human lysozyme behavior in different ambient conditions, such as in the presence of SDS surfactants and at different temperatures, may aid in providing insight into the molecular mechanisms of amyloid fibril formation and diseases associated with amyloid proteins.

Human lysozyme, a small mixed α/β protein, has been extensively studied as a model for molecular interactions with chemical surfactants, such as SDS, and for protein folding/unfolding. The molecular structure consists of 39% α-helices and 7% β-sheets. Artymiuk and Blake [30] showed that the native conformation of lysozyme has four main α-helix secondary structures: residues 5–14 (H1), 25–36 (H2), 90–100 (H3), and 110–115 (H4), with corresponding sequences of RCELARTLKR, LANWMCLAKWES, ADAVACAKRVV, and VAWRNR, respectively. SDS is known as a stabilizer for α-helical structures at high concentrations (CMC or higher) because it provides hydrophobic conditions for such secondary structures [1, 67]. The CMC value for SDS in aqueous solution ranges from 7–8 mM, which is approximately equivalent to 60 molecules of SDS, whereas our simulations contained approximately 200 SDS molecules. Therefore, in our simulations, human lysozyme was expected to maintain its secondary structure in aqueous SDS solutions. The DSSP analyses indicated that the α-helix structures were nearly completely stable during the 500000 ps MD simulation at 300 K (Fig 3). However, the secondary structures at 370 K were almost entirely disrupted, possibly due to the relatively low melting point of human lysozyme, which is 367 K [68]. Earlier experimental studies have shown that globular proteins, such as lysozyme, lose their native α/β secondary structures and gain β-sheet structural elements, thereby inducing amyloid fibril formation with increasing temperatures [69, 70], and adding SDS denaturants at sub-micellar concentrations (< CMC) further promotes this destabilization [1]. Moreover, different ambient conditions, such as high temperatures, have been reported to cause partial unfolding of protein structures [71–73]. Khan et al. indicated that when proteins were partially unfolded, SDS could potentially induce amyloid fibril formation [42]. In good agreement with these observations, the present study showed that SDS molecules at high temperatures induced α-helix-to-β-sheet transitions in human lysozyme. These structural transitions are known to be features of amyloid fibrils [74–76]. However, SDS at 300 K had fewer effects on the secondary structure of human lysozyme (Fig 3 and Table 2). Due to the high concentration of SDS surfactants, the surface of the protein was saturated with SDS molecules [46]. Subsequently, hydrophobic interactions between the protein and the SDS surfactants predominated [57]. Therefore, the SDS molecules provided a hydrophobic core for lysozyme, and the α-helices maintained their structures above the CMC of the SDS surfactants [62, 65].

Fig 3 shows that in the pure water 370 K simulation, the H4 helix transformed into a β-sheet structure over the 500000 ps simulation. Other helical structures would have likely transformed into β-sheets as the simulation time increased. The simulation results also revealed that SDS at concentrations higher than its CMC maintained the helical conformation of human lysozyme at 300 K, whereas at 370 K, most α-helix structures were lost as more β-sheet structures formed. The results during the 500000 ps simulation suggested that the SDS surfactants at high temperature could not stabilize the helical structures of human lysozyme; this would likely lead to amyloid fibril formation. As mentioned earlier, the β-sheet structures are more resistant to unfolding than the α-helix structures. The β-sheet structures would have likely maintained their structures for longer MD simulation times. This would have likely resulted in the aggregation of human lysozyme to form β-amyloid fibrils. Earlier experimental studies have shown that the α-helices and β-sheets of SDS-denatured lysozyme exhibited considerable rigidity and flexibility, respectively [29]. As shown in Fig 3 at 300 K, most α-helices maintained their secondary structure. The β-sheet structures at some points during the simulation lost and regained their structure, especially at 370 K. Therefore, significant changes in the secondary structure of the protein were observed with increasing temperature. The negatively charged head groups of SDS tended to form a stable salt bridge with the positively charged side chains of cationic amino acids [77]. The subsequent polar binding suggested that, although the hydrophobic residues have an essential role in the absorption of human lysozyme onto the SDS surfactant, the hydrophilic residues, especially Lys-1 and Arg-41 in SDS at 300 K and Arg-10, Lys-33, and Arg-62 in SDS at 370 K, favorably enhanced the polar binding energies. This suggested electrostatic interactions with the SDS sulfate head groups (please refer to Eq 7).

## Conclusions

In conclusion, our results revealed that SDS surfactants play a negative role in affecting the structural integrity of globular proteins with increasing temperature. We also found that human lysozyme is surrounded by the hydrophobic tails of SDS surfactants at SDS concentrations above its CMC. Our simulation results revealed that SDS maintains the helical conformation of human lysozyme at 300 K, whereas at 370 K, most helical structures were lost and the β-sheet content increased. Although SDS surfactants could induce the formation of amyloid fibril proteins at concentrations lower than its CMC, our results suggested that further experiments may be necessary to fully evaluate the role of SDS surfactants in the formation of β-amyloid fibrils by human lysozyme at concentrations above the CMC and at high temperatures.
